# Burnout among veterinarians working with exotic animals in Italy: a cross-sectional study using the burnout assessment tool (BAT-12)

**DOI:** 10.3389/fvets.2026.1951444

**Published:** 2026-09-03

**Authors:** Samuele Noè, Matteo Serpieri, Paola Gatti, Matteo Cesare Stucchi, Giacomo Garzaro, Mitzy Mauthe von Degerfeld

**Affiliations:** 1 Department of Public Health Sciences and Pediatrics, Occupational Medicine Unit, University of Turin, Turin, Italy; 2 Department of Veterinary Sciences, University of Turin, Grugliasco, Italy; 3 Department of Psychology, University of Milano-Bicocca, Milan, Italy; 4 Department of Medical Sciences, University of Turin, Turin, Italy

**Keywords:** burnout, exotic animals, occupational health, occupational stress, veterinarians

## Abstract

Burnout is an occupational phenomenon resulting from poorly managed chronic workplace stress, and veterinary medicine is among the professions at highest risk. Veterinarians working with exotic animals represent a specialised professional subgroup whose wellbeing may affect the availability of dedicated care for non-traditional species. To our knowledge, no study has examined burnout in this population. This study estimated burnout prevalence and identified demographic, occupational, and psychosocial work factors associated with burnout in Italian veterinarians specialising in exotic animals, within the Job Demands-Resources framework. In this cross-sectional study, 190 professionals completed an online questionnaire including the Italian 12-item Burnout Assessment Tool (BAT-12), demographic and occupational variables, and organisational and psychosocial work factors. Burnout risk was classified using pooled European BAT reference thresholds, which distinguish “no burnout,” “at risk,” and “severe burnout” categories. Associations with the total BAT-12 score were assessed using non-parametric tests, Spearman correlations, and multivariable ordinary least-squares regression. Overall, 45.3% of participants scored at or above the at-risk threshold, and 26.3% fell in the red category for severe burnout. Exhaustion was the most pronounced BAT-12 dimension. Client conflict showed the strongest independent positive association with burnout, whereas alignment with personal values, work-life balance, job autonomy, and number of children were associated with lower burnout scores. Workload intensity, age, and experience were not significantly associated with burnout. Burnout risk was frequent in this sample of Italian veterinarians specialising in exotic animals. The findings suggest that prevention strategies should address the qualitative and relational dimensions of veterinary work alongside workload management.

## 1 Introduction

Burnout is recognised by the World Health Organization in the 11th Revision of the International Classification of Diseases (ICD-11) as an occupational phenomenon resulting from chronic workplace stress that has not been successfully managed, and is listed among factors influencing health status or contact with health services rather than as a medical condition (1, 2). Veterinary medicine is among the professions at highest risk, with several survey-based studies reporting moderate-to-high burnout levels among veterinarians (3–7). Burnout also has important professional and public health consequences, including increased suicide risk, premature exit from the profession, and substantial economic costs (5, 8, 9).

Burnout in veterinary practice arises from the interaction between intrinsic stressors, such as exposure to animal suffering, euthanasia, ethical dilemmas, and client incivility, and structural demands, including long working hours, on-call shifts, administrative overload, and team conflict (6, 10, 11). According to the Job Demands-Resources (JD-R) theory, occupational wellbeing depends on the balance between job demands, requiring sustained physical, cognitive, or emotional effort, and job resources, which support goal attainment, motivation, and recovery (12, 13). Burnout primarily develops through a health-impairment process driven by excessive demands, whereas resources such as autonomy, social support, role clarity, recognition, and value alignment may buffer their impact (12–15).

Reliable measurement of burnout remains challenging. The Maslach Burnout Inventory lacks clinically validated cutoffs, uses different dimensional labels across versions and occupational groups, and omits cognitive symptoms (16). The Burnout Assessment Tool (BAT) addresses these limitations by conceptualising burnout as a higher-order construct encompassing exhaustion, mental distance, cognitive impairment, and emotional impairment (16). The Italian BAT-12 has demonstrated adequate psychometric properties (17), and clinically validated cutoff points are available from a pooled European reference sample (18).

Despite growing Italian literature on veterinary wellbeing (18, 19), no published study has applied the BAT to Italian veterinarians or specifically investigated those working with exotic animals. This subgroup represents a highly specialised professional community in which reduced wellbeing or professional attrition may affect the availability of care for species requiring dedicated expertise and facilities. Exotic animal practice forms part of zoological medicine, encompassing non-traditional companion animals, zoo animals, and wildlife (20). In this study, “exotic animals” refers to non-traditional companion animals and captive or managed wild species, including small mammals, birds, reptiles, amphibians, ornamental fish, wildlife, and zoo species. Compared with conventional companion-animal practice, this field combines heterogeneous species, rapidly evolving knowledge, limited species-specific evidence, complex anaesthetic and restraint procedures, and frequent clinical uncertainty (20, 21). It also intersects with animal welfare, wildlife management, conservation, zoonotic risk communication, and public health, making communication with owners or external collaborators particularly demanding, especially when prognosis, costs, welfare constraints, or standards of care are difficult to explain (22, 23). Dedicated studies on veterinarians working with exotic and wild species are essentially absent from the peer-reviewed literature, making this subgroup a useful model to examine whether burnout is more closely associated with workload intensity or with qualitative aspects of work, including relational conflict, clinical uncertainty, autonomy, work-life balance, and value alignment.

Guided by the JD-R framework, the present study considered client conflict, emergency duties, clinical uncertainty, and time pressure as job demands, whereas social support, autonomy, role clarity, recognition, work-life balance, and alignment with personal values were considered job resources (10–15, 20, 21). We hypothesised that burnout would be frequent in this professional group and that work-related demands, particularly client conflict and emergency duties, would be positively associated with BAT-12 total scores, whereas organisational, relational, and value-related resources would be inversely associated with BAT-12 total scores.

The aim of the present study was to assess burnout prevalence among Italian veterinarians specialising in exotic animals using the BAT-12 and the cutoff points proposed by Schaufeli and colleagues, and to test JD-R-informed hypotheses on the demographic, occupational, and psychosocial factors associated with BAT-12 total burnout severity.

## 2 Materials and methods

### 2.1 Study design and ethics

This cross-sectional study assessed burnout among Italian veterinarians specialising in exotic animals (exotic pets, wildlife, and zoo species). The study was approved by the Bioethics Committee of the University of Turin (protocol no. 0348953/2025). All participants provided informed consent, including authorisation for the scientific use of anonymised data.

### 2.2 Participants and recruitment

The target population comprised approximately 600 veterinary professionals practising exotic animal medicine in Italy, estimated from membership data of the Società Italiana Veterinari Animali Esotici (SIVAE; Italian Society of Exotic Animal Veterinarians). No formal *a priori* sample size calculation was performed. Given the relatively small and well-defined target population, the study adopted a maximal-recruitment approach, seeking to include as many eligible participants as possible during the data-collection period. Participants completed an anonymous online questionnaire administered through Google Forms. The survey was launched during the SIVAE National Meeting in October 2025, where the study was introduced orally and a QR code linking to the questionnaire was displayed. Two reminder emails containing the survey link were sent to SIVAE members in November 2025 and January 2026. The survey was presented again during the SIVAE National Meeting in March 2026, using the same oral introduction and QR code, and remained open until May 2026. Participation was voluntary and unremunerated. To preserve anonymity, participants were not required to log in or provide account identifiers, and no technical restriction on multiple submissions was applied. Responses were manually screened for potential duplicate submissions, and both during conference presentations and in reminder emails participants were explicitly instructed not to complete the questionnaire again if they had already participated. Only complete BAT-12 responses were included in the analysis.

### 2.3 Measures

Primary outcome: the Burnout Assessment Tool (BAT-12). Burnout was assessed using the validated Italian 12-item Burnout Assessment Tool (BAT-12), which measures four core dimensions: exhaustion, mental distance, emotional impairment, and cognitive impairment. Items are rated on a 5-point Likert scale (1 = “never”; 5 = “always”). Dimension and total scores were calculated as the mean of the corresponding items (range 1–5), with higher scores indicating greater burnout. The BAT-12 total score was used as the primary inferential outcome because the BAT conceptualises burnout as an overall syndrome comprising four interrelated dimensions and because the available risk thresholds are defined for the total score. Subscale scores were used descriptively to characterise the burnout profile. Risk categories followed the pooled European BAT cutoffs (24): total scores < 2.54 indicated no burnout (green), scores from 2.54 to 2.95 indicated an at-risk profile (orange), and scores ≥ 2.96 indicated severe burnout (red).

Demographic and occupational variables. A structured questionnaire collected demographic data (age, sex, relationship status, number of children) and occupational characteristics (years of professional experience, main field of activity, work setting, weekly working hours, monthly night shifts, monthly holiday shifts, monthly on-call nights, emergency duties, employment type, and prevalent animal category). Geographic origin was grouped into three macro-areas (North, Centre, South). The complete questionnaire is provided as Supplementary Appendix 1, 2.

Organisational and psychosocial work factors. Twelve single-item measures assessed organisational and psychosocial working conditions using the same 5-point Likert scale: colleague support, supervisor support, client conflict, work-life balance, career development opportunities, recognition of effort, job autonomy, sustainable workload, role clarity, involvement in decision-making, alignment with personal values, and remuneration satisfaction. These items operationalised JD-R work demands and resources in this professional context. Their use as single-item indicators reduced respondent burden and questionnaire length but may have provided less precise measurement than validated multi-item scales. Eleven items represented job resources, with higher scores indicating more favourable working conditions. Client conflict represented a job demand, with higher scores indicating greater perceived conflict.

### 2.4 Statistical analysis

All analyses were performed in Python (pandas, NumPy, SciPy, pingouin, statsmodels, matplotlib, and seaborn). Internal consistency of the BAT-12 and its subscales was evaluated using Cronbach’s alpha. Continuous variables are reported as mean ± standard deviation (SD) and median with interquartile range (IQR), and categorical variables as counts and percentages. Because the BAT-12 total score was not normally distributed (Shapiro–Wilk test), non-parametric methods were used for bivariate analyses.

Associations between the BAT-12 total score and ordinal or continuous variables were assessed using Spearman’s rank correlation coefficient (ρ). Between-group comparisons used the Mann–Whitney U test (two groups) or Kruskal-Wallis test (three or more groups). Independent correlates of burnout were examined using a multivariable ordinary least-squares (OLS) regression model with standardised coefficients (β). Candidate predictors were selected based on theoretical relevance within the JD-R framework and evidence of bivariate association with the BAT-12 total score. To limit overfitting, the final model retained a restricted set of predictors representing key work demands, job resources, and relevant demographic/family variables. Multicollinearity was assessed using the variance inflation factor (VIF), and model fit by the adjusted R^2^, Akaike information criterion (AIC), and Bayesian information criterion (BIC). Missing values were not imputed. Descriptive statistics and bivariate analyses were calculated using the available observations for each variable, whereas the multivariable regression used complete cases across the variables included in the model. Some organisational and psychosocial items were not mandatory because their applicability depended on the respondent’s working arrangement. The remuneration-satisfaction item was introduced after data collection had commenced and was therefore available only for a subset of participants; this variable was not included in the final multivariable model. Statistical significance was set at *p* < 0.05 (two-sided).

## 3 Results

Complete BAT-12 responses were obtained from 190 veterinarians. Completion of the organisational and psychosocial items ranged from 92.1 to 100%, except for remuneration satisfaction. This item was introduced after data collection had commenced and was completed by 108 participants (56.8%). Analyses involving this variable were therefore based on these available responses.

The sample was predominantly female (62.1%), aged 35–49 years (49.5%), in a relationship (75.3%), and without children (60.5%). Most participants had 6–30 years of professional experience (82.1%), worked 30–40 h per week (42.6%), and were self-employed (93.7%). Approximately half of the participants (49.5%) performed emergency duties, whereas most did not perform night shifts (85.3%). Almost all respondents worked with exotic pets (98.4%), with a minority also treating wildlife and/or zoo or aquarium species (14.2%). Most practised in Northern Italy (61.6%), primarily in outpatient clinics or veterinary clinics. Detailed sample characteristics are presented in Supplementary Table 1.

### 3.1 Scale reliability and burnout scores

**Figure 1:**
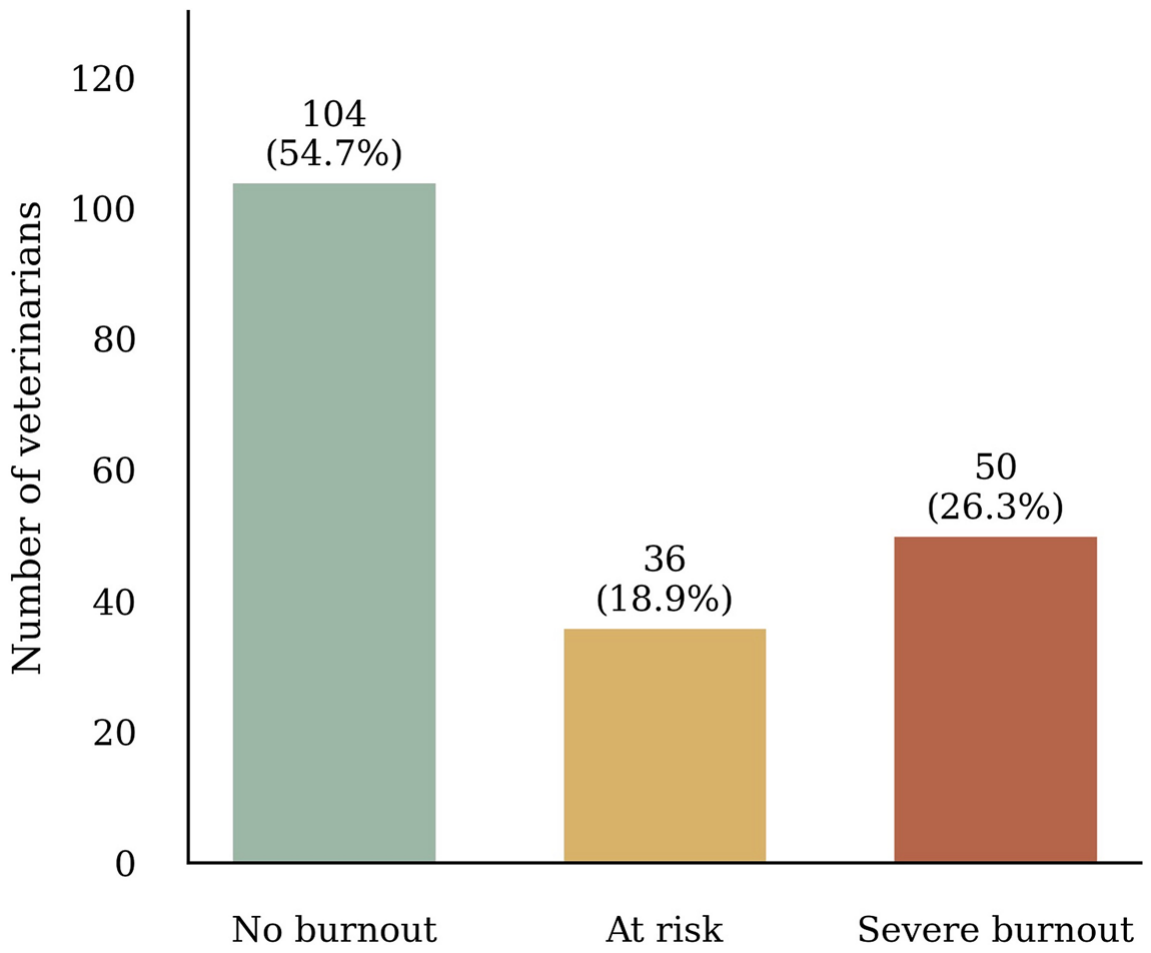
Distribution of participants across BAT-12 burnout categories according to the pooled European cut-off points proposed by Schaufeli et al. (24). No burnout: BAT-12 total score < 2.54; At risk: 2.54–2.95; Severe burnout: ≥ 2.96. Values above bars indicate number and percentage of participants in each category.

The BAT-12 showed good internal consistency (Cronbach’s α = 0.88), with acceptable to good reliability across all subscales (Table 1). The mean total burnout score was 2.52 ± 0.65 (median 2.50, IQR 2.02–3.00). Among the four dimensions, exhaustion had the highest mean score (3.27 ± 0.78), followed by mental distance, emotional impairment, and cognitive impairment (Table 1; Supplementary Figure 1). The total score departed from normality (Shapiro–Wilk test: W = 0.985, *p* = 0.039), supporting the use of non-parametric analyses. Based on the pooled European cutoffs (24), 54.7% of participants were classified as no burnout, 18.9% as at risk, and 26.3% as severe burnout; overall, 45.3% scored at or above the at-risk threshold (Figure 1).

**Table 1:** Internal consistency, descriptive statistics, and burnout risk prevalence of the BAT-12 (*N* = 190).

BAT-12 score	α	Mean ± SD	Median (IQR)
Total burnout	0.88	2.52 ± 0.65	2.50 (2.02–3.00)
Exhaustion	0.81	3.27 ± 0.78	3.33 (2.67–3.67)
Mental distance	0.76	2.47 ± 0.94	2.33 (1.67–3.00)
Emotional impairment	0.83	2.28 ± 0.92	2.17 (1.67–3.00)
Cognitive impairment	0.81	2.04 ± 0.70	2.00 (1.67–2.67)
Risk category (total score)	n	%	
Green—no burnout (< 2.54)	104	54.7	
Orange—at risk (2.54–2.95)	36	18.9	
Red—severe burnout (≥ 2.96)	50	26.3	

α, Cronbach’s alpha; SD, standard deviation; IQR, interquartile range (25th-75th percentile). The α reported for the total burnout row refers to the full 12-item scale. Scores range from 1 to 5. Cut-off points are from Schaufeli et al. (24), pooled European sample.

### 3.2 Organisational and psychosocial work factors

Mean scores for the organisational and psychosocial work factors are summarised in Table 2. The highest scores were observed for alignment with personal values, involvement in decision-making, and role clarity, whereas the lowest concerned work-life balance and remuneration satisfaction. The client conflict item indicated a moderate level of perceived tension in relationships with clients or external collaborators.

**Table 2:** Descriptive statistics of the organisational and psychosocial work factors (1–5 Likert scale).

Work factor	Mean ± SD	Median	N
Personal values alignment	3.65 ± 1.03	4.0	190
Involvement in decisions	3.52 ± 1.22	4.0	187
Role clarity	3.51 ± 1.16	4.0	184
Colleague support	3.34 ± 1.19	3.0	189
Supervisor support	3.09 ± 1.36	3.0	175
Job autonomy	3.05 ± 1.10	3.0	190
Recognition of effort	2.89 ± 1.01	3.0	190
Career development	2.85 ± 1.07	3.0	190
Client conflict *	2.76 ± 1.00	3.0	189
Sustainable workload	2.67 ± 1.06	3.0	190
Remuneration satisfaction ^†^	2.51 ± 1.23	2.0	108
Work-life balance	2.23 ± 1.14	2.0	190

Items are ordered by descending mean. SD, standard deviation. *Client conflict represents a job demand, with higher scores indicating greater perceived conflict; for all resource items, higher scores indicate more favourable working conditions. † The remuneration satisfaction item was introduced after data collection had commenced and was therefore available for 108 participants.

### 3.3 Bivariate correlates of burnout

**Figure 2:**
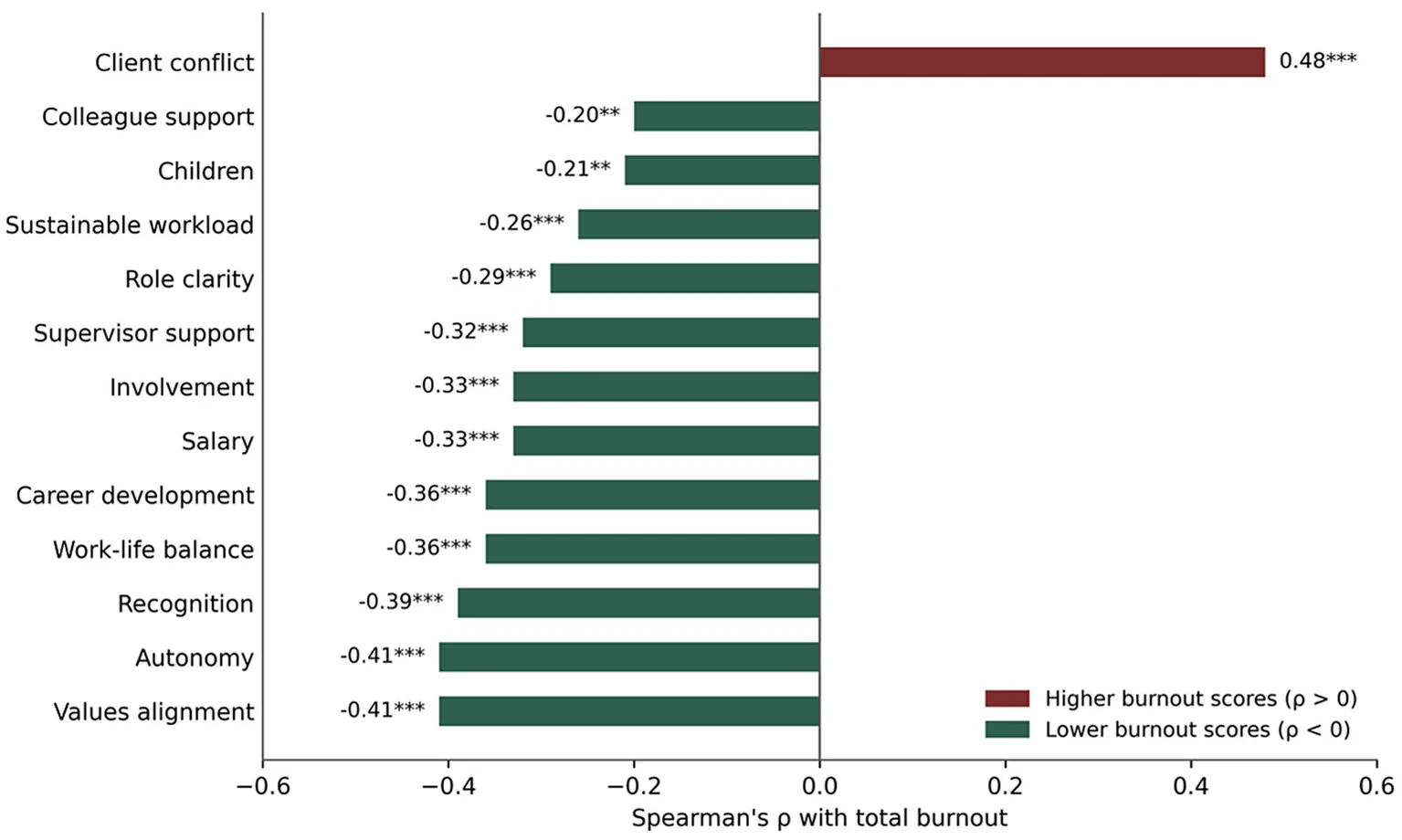
Spearman correlations between the BAT-12 total score and demographic, occupational, and organisational factors. Horizontal bars ordered by coefficient; positive values indicate higher burnout scores, whereas negative values indicate lower burnout scores. *** *p* < 0.001; ** *p* < 0.01.

Spearman correlations between the BAT-12 total score and the hypothesised demographic, occupational, and organisational factors are shown in Figure 2. Client conflict showed the strongest positive association with burnout (ρ = 0.48, *p* < 0.001). The strongest inverse associations were observed for personal values alignment (ρ = −0.41), job autonomy (ρ = −0.41), recognition of effort (ρ = −0.39), work-life balance (ρ = −0.36), career development (ρ = −0.36), remuneration satisfaction (ρ = −0.33), involvement in decisions (ρ = −0.33), and supervisor support (ρ = −0.32; all *p* ≤ 0.001). Additional inverse associations were observed for role clarity, sustainable workload, number of children, and colleague support. No significant associations were found for workload-intensity variables (night shifts, weekly hours, or on-call nights), age, or years of professional experience (all *p* > 0.05).

### 3.4 Between-group comparisons

The only statistically significant between-group difference concerned emergency duties: veterinarians performing emergency activities reported higher burnout scores than those who did not (median 2.58 vs. 2.33; Mann–Whitney U = 3660.0, *p* = 0.025). No significant differences were detected by sex, main field of activity, geographic macro-area, employment type, or relationship status (all *p* > 0.05). Full comparisons are provided in Supplementary Table 2.

### 3.5 Multivariable analysis

**Figure 3:**
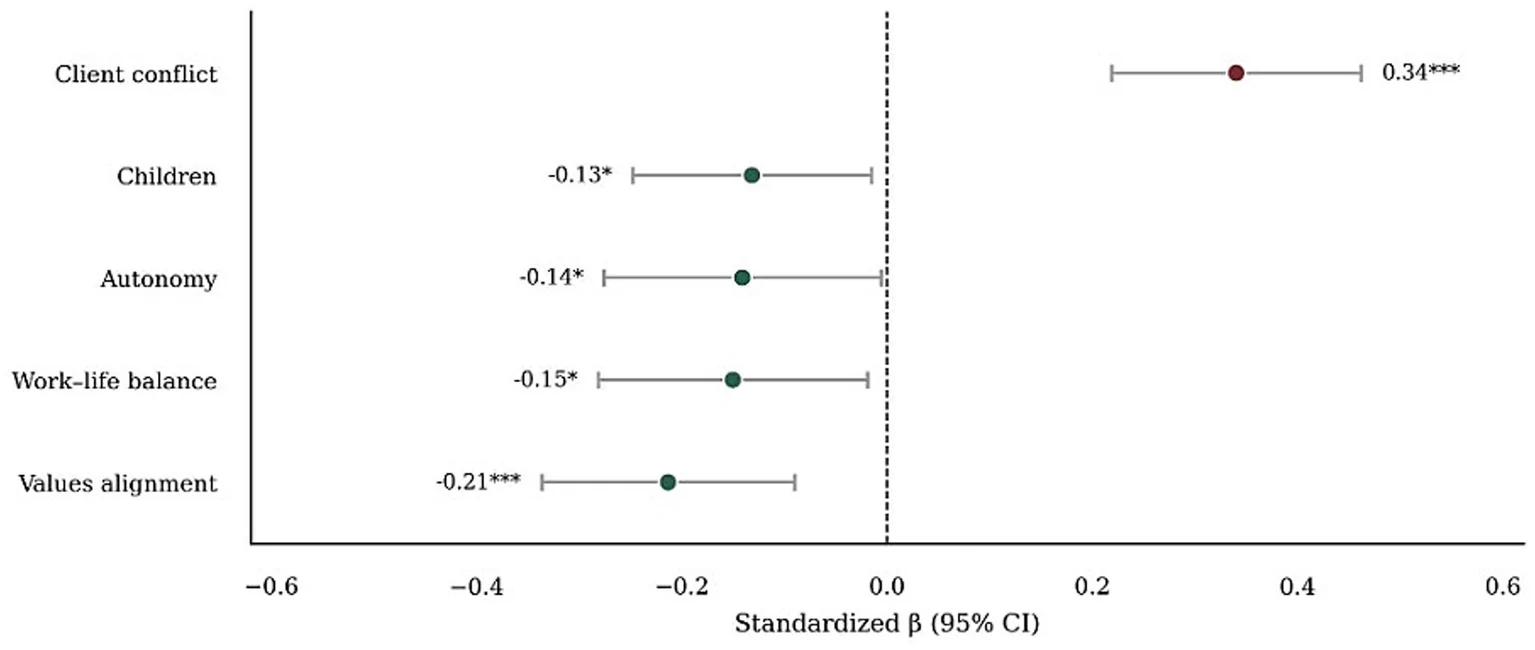
Forest plot of standardised coefficients (*β*) from the multivariable OLS regression model for the BAT-12 total score (*N* = 189). Points are *β* estimates with 95% confidence intervals; the dashed line marks *β* = 0. Model adjusted *R*^2^ = 0.38; maximum *VIF* = 1.42.

A multivariable OLS regression model identified five independent correlates of the total burnout score (Figure 3). The model explained a substantial share of the variance (adjusted R^2^ = 0.38; R^2^ = 0.40; F-test *p* < 0.001) with acceptable multicollinearity (maximum VIF = 1.42). Client conflict was the strongest independent positive correlate of burnout (*β* = 0.34, *p* < 0.001), whereas personal values alignment (*β* = −0.21, *p* < 0.001), work-life balance (*β* = −0.15, *p* = 0.025), job autonomy (*β* = −0.14, *p* = 0.040), and number of children (*β* = −0.13, *p* = 0.027) were independently associated with lower burnout scores.

## 4 Discussion

This study assessed burnout among Italian veterinarians specialising in exotic animals using the BAT-12 within a Job Demands-Resources (JD-R) analytical framework. Three findings stand out. First, 45.3% of the 190 respondents scored at or above the orange “at-risk” threshold proposed by Schaufeli and colleagues, and 26.3% fell within the red “severe burnout” category (24). Second, exhaustion was the most pronounced BAT-12 dimension. Third, the multivariable analysis identified client conflict as the strongest independent positive correlate of burnout (*β* = 0.34), with alignment with personal values, work-life balance, job autonomy, and number of children associated with lower burnout scores. Together, these findings suggest that, in this professional group, relational and value-based dimensions of work may be more informative than classical workload-intensity variables in explaining occupational distress.

The predominance of exhaustion is consistent with its role as the core energetic component of burnout, reflecting the depletion of mental and physical resources after sustained exposure to work-related demands (16). Its predominance in our sample suggests that burnout may be expressed primarily as energetic depletion rather than as disengagement or impaired emotional and cognitive functioning. This interpretation is consistent with the JD-R health-impairment process (12, 13) and may be particularly relevant in exotic animal practice, where clinical uncertainty, heterogeneous species, complex case management, communication with owners or external collaborators, and welfare-related decision-making impose sustained demands (20–23).

The prevalence of severe burnout observed in our sample (26.3%) was numerically lower than the 33.6% reported in the only previous BAT-based veterinary study conducted in Spain by Osca and colleagues (25), and numerically lower than the 50.2% of high burnout documented by Ouedraogo and colleagues in 5,020 United States veterinarians measured with the Maslach Burnout Inventory (3). These comparisons are descriptive and should be interpreted cautiously because the studies differed in population characteristics, sampling strategy, and burnout measurement. Osca et al. used the longer BAT-C in a large and heterogeneous national sample of Spanish veterinarians, whereas the present study used the BAT-12 in a smaller and more specialised subgroup. Nevertheless, both studies support the applicability of the BAT framework to veterinary populations despite differences in instrument version, sampling strategy, and professional composition (25).

When the at-risk band is included, 45.3% of participants in this sample fell within an elevated burnout-risk range, consistent with broader evidence that burnout represents a substantial occupational concern in veterinary medicine (4, 6, 7, 9).

The prevalence observed in this sample does not suggest that veterinarians working with exotic animals experience higher overall burnout than other veterinary populations. Rather, the specific context of exotic animal practice may help explain the pattern of burnout observed within this group, particularly the predominance of exhaustion and the relevance of relational demands. These findings may reflect the energetic cost of clinically uncertain, communicatively demanding, and welfare-sensitive work situations. Clinical uncertainty may become particularly salient during interactions with owners or external collaborators, especially when diagnostic limitations, prognosis, costs, or welfare constraints must be discussed (20, 21).

The observed pattern of correlates is consistent with the JD-R model (12, 13). Among the demands, client conflict emerged as the strongest positive correlate of burnout, in line with previous Italian (11) and international (10) evidence that the veterinarian-client relationship is one of the most demanding aspects of contemporary veterinary practice. In exotic animal practice, this association may be amplified by the gap between owners’ emotional investment and the limited availability of standardised diagnostic or therapeutic pathways for many species (20, 21). Owners may have limited species-specific knowledge, while veterinarians must communicate uncertainty, costs, welfare constraints, and sometimes poor prognosis. This combination may increase disagreement and help explain why client conflict was more strongly associated with burnout than workload-intensity variables (22, 23).

Notably, none of the workload-intensity variables, including weekly working hours, night shifts, on-call frequency, age, or years of professional experience, was significantly associated with burnout. These findings suggest that qualitative characteristics of work, particularly its emotional and relational demands, may be more important than workload quantity. Emergency duties were the only workload-related variable associated with burnout, possibly reflecting the cumulative strain of an unpredictable caseload rather than time exposure per se. Similarly, accumulated professional experience may be insufficient to reduce exposure to uncertainty, client conflict, or emergency-related strain in this specialised field.

Among the resources, alignment with personal values stood out as the most robust correlate of lower burnout scores (*β* = −0.21), consistent with the broader literature on the role of meaningful work in mitigating exhaustion (12) and with Italian evidence on the ethical dimensions of veterinary practice (11). In exotic animal medicine, alignment with personal values may be particularly relevant because the perceived meaning and purpose of work are often linked to animal welfare, species conservation, and expertise in a niche clinical area (20).

The inverse association between number of children and burnout was unexpected and should be interpreted cautiously, particularly because the subgroup with more than two children was small. Nevertheless, the association remained significant after adjustment, suggesting that it was not fully explained by age or other included covariates. One possible interpretation is that family responsibilities may provide alternative sources of meaning and social identity or reduce the centrality of professional stressors. Conversely, veterinarians with more stable working conditions or greater control over their professional life may be more likely to have children. Longitudinal studies are needed to clarify the direction of this association.

Several features of the population deserve attention. The predominance of self-employed practitioners (93.7%) is an important characteristic of this sample and may limit direct comparison with veterinary populations in which employed practitioners predominate. Self-employment may partly explain the relevance of autonomy and personal values alignment, as greater control over clinical and organisational decisions may coexist with greater exposure to client expectations, economic pressure, administrative responsibilities, and business-related demands. Consequently, the pattern of associations observed here may not fully generalise to employed veterinarians working within more structured organisational settings. The absence of significant associations between burnout and demographic variables such as sex, age, or geographic macro-area contrasts with previous Italian (11) and Spanish (25) studies reporting higher burnout levels among female veterinarians. One possible explanation is that, in this specialised field, burnout may be driven more by shared characteristics of the work itself than by demographic differences. Client conflict, clinical uncertainty, emergency duties, and the need to preserve professional meaning may affect veterinarians across demographic groups. Furthermore, the relatively small size of this professional community may limit opportunities for peer exchange, informal support, and workload redistribution, increasing the impact of work-related pressures. The sample included 190 participants, corresponding to approximately one-third of the estimated target population of about 600 veterinary professionals practising exotic animal medicine in Italy. However, because comprehensive demographic data for the entire target population are not available, formal representativeness could not be assessed. Moreover, the voluntary convenience sampling strategy may have introduced selection bias. The findings should therefore be interpreted as observations from the study sample rather than assumed to generalise to all Italian veterinarians working with exotic animals.

Several limitations should be acknowledged. The cross-sectional design does not allow causal inferences on the direction of the observed associations. In addition, the convenience sampling strategy and voluntary survey design may have favoured participation by veterinarians more sensitive to wellbeing issues or, conversely, excluded those too exhausted or disengaged to participate, potentially affecting prevalence estimates in either direction. The sample size (*n* = 190), while substantial relative to the estimated population of Italian exotic animal veterinarians (~600), limits statistical power for subgroup analyses. Because no account login or unique participant identifier was collected to preserve anonymity, duplicate participation could not be completely excluded, although participants were explicitly instructed not to submit more than one response and responses were manually screened. The pooled European cutoff points proposed by Schaufeli and colleagues were adopted in the absence of Italian veterinary-specific validation, and although these cutoffs have demonstrated cross-cultural robustness (24), their transferability to the Italian veterinary context warrants confirmation. Although guided by the JD-R framework, the present study examined only direct associations with burnout severity and did not test demand-resource interactions or buffering effects. The use of single-item indicators for organisational demands and resources may also have limited measurement precision compared with validated multi-item instruments. Because remuneration satisfaction was assessed only in participants completing the later version of the questionnaire, its association with burnout was estimated in a smaller subset and may be affected by differences between recruitment phases. This finding should therefore be interpreted cautiously. Future longitudinal studies should examine these mechanisms and determine whether resources such as autonomy, work-life balance, and value alignment moderate the impact of demands including client conflict and emergency duties.

Future research should expand on these findings through longitudinal designs capable of clarifying the temporal relationships underlying veterinary burnout, the development of Italian veterinary-specific BAT cutoff points, and comparisons with other veterinary specialisations. The integration of personal resources and the systematic measurement of moral distress, already documented in Italian veterinary populations (11), would further enrich the conceptual model.

From an applied perspective, these findings support targeted veterinary client-communication training together with strategies to strengthen professional support within this specialised community. For veterinarians working with exotic animals, such training may be particularly relevant because clinical uncertainty, welfare constraints, costs, and prognosis are often difficult to communicate when species-specific knowledge and standardised care pathways are limited. Communication training should therefore include expectation management, communication of uncertainty, conflict de-escalation, and discussion of welfare-related limits to care. Such approaches are consistent with emerging intervention evidence in veterinary healthcare teams (26) and could be incorporated into undergraduate, postgraduate, and continuing professional training (27). Organisational strategies promoting autonomy, work-life balance, value alignment, and peer support may also help reduce burnout risk in this specialised professional community.

## 5 Conclusion

Burnout was a substantial occupational health concern in this sample of Italian veterinarians specialising in exotic animals, with nearly one in two participants scoring at or above the at-risk threshold and one in four meeting the criterion for severe burnout (24). Client conflict emerged as the principal correlate of burnout, whereas alignment with personal values, work-life balance, and job autonomy were associated with lower burnout scores. These findings suggest that qualitative and relational aspects of veterinary work may be more relevant than workload intensity in shaping occupational distress, supporting interventions focused on client communication, conflict management, and organisational resources, while highlighting the need for Italian veterinary-specific BAT cutoff validation and longitudinal research.

## Data Availability

The original contributions presented in the study are included in the article/Supplementary material, further inquiries can be directed to the corresponding author.
