# Supplementary material for: Burnout among veterinarians working with exotic animals in Italy: a cross-sectional study using the burnout assessment tool (BAT-12)
Source: Front Vet Sci. 2026 Sep 3;13:1951444. doi: 10.3389/fvets.2026.1951444 (PMC13582189; doi:10.3389/fvets.2026.1951444)
Supplement: Supplementary file 1 [file Table_1.DOCX]

Supplementary Material

**Supplementary Table 1.** Demographic and occupational characteristics of the sample (N = 190). *Participants could select more than one animal category; therefore, percentages do not sum to 100%.

| **Characteristic** | **n** | **%** |
| --- | --- | --- |
| **Sex** | | |
| Female | 118 | 62.1 |
| Male | 69 | 36.3 |
| Not reported | 3 | 1.6 |
| **Age band (years)** | | |
| ≤ 29 | 13 | 6.8 |
| 30-34 | 27 | 14.2 |
| 35-49 | 94 | 49.5 |
| 50-59 | 46 | 24.2 |
| 60-69 | 10 | 5.3 |
| **Relationship status** | | |
| In a relationship | 143 | 75.3 |
| Single | 47 | 24.7 |
| **Children** | | |
| None | 115 | 60.5 |
| 1 | 33 | 17.4 |
| 2 | 35 | 18.4 |
| > 2 | 7 | 3.7 |
| **Years of professional experience** | | |
| ≤ 5 | 22 | 11.6 |
| 6-15 | 74 | 38.9 |
| 16-30 | 82 | 43.2 |
| > 30 | 12 | 6.3 |
| **Weekly working hours** | | |
| < 30 | 25 | 13.2 |
| 30-40 | 81 | 42.6 |
| 41-50 | 56 | 29.5 |
| > 50 | 28 | 14.7 |
| **Monthly night shifts** | | |
| None | 162 | 85.3 |
| < 3 | 16 | 8.4 |
| 3-6 | 8 | 4.2 |
| > 6 | 4 | 2.1 |
| **Emergency duties** | | |
| Yes | 94 | 49.5 |
| No | 96 | 50.5 |
| **Employment type** | | |
| Self-employed | 178 | 93.7 |
| Both | 9 | 4.7 |
| Employee | 3 | 1.6 |
| **Main field of activity** | | |
| Multidisciplinary / mixed | 121 | 63.7 |
| Pure clinical practice | 69 | 36.3 |
| **Type of animals treated*** |  |  |
| Exotic pets | 187 | 98.4 |
| Wildlife | 20 | 10.5 |
| Zoo / aquarium species | 9 | 4.7 |
| **Geographic macro-area** | | |
| North | 117 | 61.6 |
| Centre | 34 | 17.9 |
| South | 39 | 20.5 |

**Supplementary Table 2**. Comparisons of the BAT-12 total score across demographic and occupational groups (non-parametric tests). MWU, Mann-Whitney U test; KW, Kruskal-Wallis test; SD, standard deviation. * p < 0.05. The employment-type comparison (Kruskal-Wallis, p = 0.371) is not tabulated because two of its three categories contained fewer than ten participants.

| **Grouping factor** |  | **n** | **Mean ± SD** | **Median (IQR)** | **Test** | **p** |
| --- | --- | --- | --- | --- | --- | --- |
| Emergency duties | Yes | 94 | 2.61 ± 0.62 | 2.58 (2.17 - 3.00) | MWU | 0.025* |
|  | No | 96 | 2.42 ± 0.66 | 2.33 (1.90 - 2.92) |  |  |
| Sex | Female | 118 | 2.54 ± 0.64 | 2.50 (2.08 - 2.92) | MWU | 0.517 |
|  | Male | 69 | 2.47 ± 0.67 | 2.33 (1.92 - 3.00) |  |  |
| Activity | Pure clinical | 69 | 2.62 ± 0.71 | 2.50 (2.08 - 3.17) | MWU | 0.194 |
|  | Multidisc./mixed | 121 | 2.46 ± 0.61 | 2.42 (2.00 - 2.92) |  |  |
| Relationship | In a relationship | 143 | 2.48 ± 0.59 | 2.33 (2.04 - 2.92) | MWU | 0.175 |
|  | Single | 47 | 2.62 ± 0.78 | 2.67 (2.04 - 3.17) |  |  |
| Macro-area | North | 117 | 2.47 ± 0.65 | 2.33 (1.92 - 3.00) | KW | 0.364 |
|  | Centre | 34 | 2.58 ± 0.49 | 2.54 (2.25 - 2.81) |  |  |
|  | South | 39 | 2.61 ± 0.74 | 2.58 (2.08 - 3.04) |  |  |

**Supplementary Figure 1**. Mean BAT-12 subscale scores. Bars indicate mean scores for each BAT-12 dimension on a 1-5 scale; error bars indicate standard deviations. Values at the end of bars indicate mean scores. Subscales are ordered by ascending mean.

**Supplementary Appendix 1. Original Italian questionnaire administered to participants**

The questionnaire was administered in Italian. Items are reported below as presented to participants, excluding the introductory information sheet, consent text, privacy information, and Google Forms interface elements.

***Dati demografici e professionali***

1. Regione
   Opzioni: Abruzzo; Basilicata; Calabria; Campania; Emilia Romagna; Friuli Venezia Giulia; Lazio; Liguria; Lombardia; Marche; Molise; Piemonte; Puglia; Sardegna; Sicilia; Toscana; Trentino Alto Adige; Umbria; Val D’Aosta; Veneto.
2. Sesso
   Opzioni: Maschile; Femminile; Preferisco non rispondere.
3. Fascia d'età
   Opzioni: ≤ 29 anni; 30 - 34 anni; 35 - 49 anni; 50 - 59 anni; 60 - 69 anni; ≥ 70 anni.
4. Relazioni
   Opzioni: Single; In coppia.
5. Figli
   Opzioni: Nessuno; 1; 2; > 2.
6. Anni di professione
   Opzioni: ≤ 5 anni; 6 - 15 anni; 16 - 30 anni; > 30 anni.
7. Ore di lavoro settimanali (media)
   Opzioni: < 30; 30 - 40; 41 - 50; > 50.
8. Attività di pronto soccorso
   Opzioni: Sì; No.
9. Festivi mensili (compresi: sabato, domenica e festivi)
   Opzioni: Nessuno; < 3; tra 3 e 6; > 6.
10. Turni notturni mensili
    Opzioni: Nessuno; < 3; tra 3 e 6; > 6.
11. Giorni di reperibilità notturna mensili
    Opzioni: Nessuno; < 5; tra 5 e 9; > 9.
12. Attività principale
    Opzioni: Clinica medica; Chirurgia / Anestesia; Riproduzione; Anatomia patologica; Diagnostica / Laboratorio; Altro.
13. Luogo di lavoro principale
    Opzioni: Ambulatorio; Clinica; Ospedale; Zoo / Bioparco / Acquario; CRAS (Centro recupero animali selvatici); Azienda Sanitaria Locale; Industria / Farmacologia; Università / Strutture di Ricerca; Libera professione senza struttura fissa; Altro.
14. Tipologia contratto di lavoro
    Opzioni: Dipendente; Autonomo / Libero Professionista; Entrambi.
15. Attività prevalente su
    Opzioni: Pet non convenzionali; Selvatici; Animali da zoo / bioparco / acquario.

***Questionario BAT-12 (versione italiana)***

Scala di risposta per tutti gli item BAT-12: 1 = Mai; 2 = Raramente; 3 = Qualche volta; 4 = Spesso; 5 = Sempre.

1. Al lavoro mi sento mentalmente esausto/a
2. Dopo una giornata di lavoro, per me è difficile recuperare le energie
3. Al lavoro mi sento fisicamente esausto/a
4. Ho difficoltà a provare un qualche entusiasmo per il mio lavoro
5. Provo una forte avversione per il mio lavoro
6. Sono scettico/a rispetto al significato che il mio lavoro ha per gli altri
7. Al lavoro mi sento incapace di controllare le mie emozioni
8. Al lavoro mi capita di arrabbiarmi o sentirmi triste senza sapere perché
9. Al lavoro mi capita di avere delle reazioni esagerate senza volerlo
10. Al lavoro faccio fatica a mantenere l'attenzione
11. Quando lavoro ho difficoltà a pensare con lucidità
12. Al lavoro faccio degli errori perché penso ad altro

***Indagine Benessere Organizzativo***

Scala di risposta per tutti gli item: 1 = Mai; 2 = Raramente; 3 = Qualche volta; 4 = Spesso; 5 = Sempre.

[Nota del modulo originale: se non applicabile (es. lavoro senza colleghi / responsabili), non rispondere agli item 1, 2, 3, 9 e 10.]

1. Quando sono in difficoltà so di poter contare sull'aiuto dei/delle miei/mie colleghi/e
2. Mi sento libero/a di discutere con i responsabili le problematiche legate al mio lavoro
3. Percepisco tensioni frequenti nelle relazioni con utenti, clienti o collaboratori esterni
4. Sento un buon equilibrio tra il tempo dedicato al lavoro e quello dedicato alle attività personali e familiari
5. Vedo opportunità concrete di sviluppo professionale
6. Sento che il mio impegno sul lavoro è ripagato dai risultati
7. Sento di poter decidere come organizzare gran parte del mio lavoro
8. Il tempo a disposizione mi è sufficiente per completare le attività con cura
9. Il mio ruolo è ben definito all'interno degli obiettivi del team e dell’organizzazione
10. Mi sento coinvolto/a nei processi decisionali che impattano sul mio lavoro
11. Ritengo che il mio lavoro sia allineato ai miei valori personali
12. Sono generalmente soddisfatto/a del mio compenso economico

**Supplementary Appendix 2. English translation of the questionnaire**

The questionnaire was administered to participants in Italian. This English version is provided for transparency and does not represent the version completed by participants. Items are reported below in the same order as in Supplementary File 1, excluding the introductory information sheet, consent text, privacy information, and Google Forms interface elements.

The BAT-12 was administered using the validated Italian version. The English wording below is provided for transparency and corresponds to the official work-related BAT item wording.

***Demographic and professional data***

1. Region
   Options: Abruzzo; Basilicata; Calabria; Campania; Emilia-Romagna; Friuli Venezia Giulia; Lazio; Liguria; Lombardy; Marche; Molise; Piedmont; Apulia; Sardinia; Sicily; Tuscany; Trentino-Alto Adige; Umbria; Aosta Valley; Veneto.
2. Sex
   Options: Male; Female; Prefer not to answer.
3. Age group
   Options: ≤ 29 years; 30-34 years; 35-49 years; 50-59 years; 60-69 years; ≥ 70 years.
4. Relationship status
   Options: Single; In a relationship.
5. Children
   Options: None; 1; 2; > 2.
6. Years in professional practice
   Options: ≤ 5 years; 6-15 years; 16-30 years; > 30 years.
7. Weekly working hours (average)
   Options: < 30; 30-40; 41-50; > 50.
8. Emergency care duties
   Options: Yes; No.
9. Monthly holiday duties, including Saturdays, Sundays, and public holidays
   Options: None; < 3; between 3 and 6; > 6.
10. Monthly night shifts
    Options: None; < 3; between 3 and 6; > 6.
11. Monthly nights on call
    Options: None; < 5; between 5 and 9; > 9.
12. Main professional activity
    Options: Internal medicine; Surgery / Anaesthesia; Reproduction; Pathology; Diagnostics / Laboratory; Other.
13. Main workplace
    Options: Outpatient practice; Veterinary clinic; Veterinary hospital; Zoo / Aquarium; Wildlife rescue centre; Local health authority; Industry / Pharmacology; University / Research facility; Freelance practice without a fixed facility; Other.
14. Employment type
    Options: Employee; Self-employed / Freelance practitioner; Both.
15. Prevalent animal category
    Options: Exotic pet animals; Wildlife; Zoo / aquarium animals.

***BAT-12 questionnaire***

Response scale for all BAT-12 items: 1 = Never; 2 = Rarely; 3 = Sometimes; 4 = Often; 5 = Always.

The BAT-12 was administered using the validated Italian version. The English item wording should follow the original BAT-12 wording provided by Schaufeli, De Witte and Desart.

1. At work, I feel mentally exhausted.
2. After a day at work, I find it hard to recover my energy.
3. Everything I do at work requires a great deal of effort.
4. I struggle to find any enthusiasm for my work.
5. I feel a strong aversion towards my job.
6. I’m cynical about what my work means to others.
7. At work, I feel unable to control my emotions.
8. At work, I may overreact unintentionally.
9. At work, I struggle to think clearly.
10. At work, I have trouble staying focused.
11. When I’m working, I have trouble concentrating.
12. I make mistakes in my work because I have my mind on other things.

***Organisational wellbeing survey***

Response scale for all items: 1 = Never; 2 = Rarely; 3 = Sometimes; 4 = Often; 5 = Always.

Note from the original form: if not applicable, for example in the absence of colleagues or supervisors, do not answer items 1, 2, 3, 9 and 10.

1. When I am in difficulty, I know I can rely on help from my colleagues.
2. I feel free to discuss work-related problems with supervisors.
3. I perceive frequent tensions in relationships with users, clients, or external collaborators.
4. I feel there is a good balance between the time devoted to work and the time devoted to personal and family activities.
5. I see concrete opportunities for professional development.
6. I feel that my effort at work is rewarded by results.
7. I feel able to decide how to organise most of my work.
8. The time available to me is sufficient to complete tasks carefully.
9. My role is well defined within the goals of the team and the organisation.
10. I feel involved in decision-making processes that affect my work.
11. I believe that my work is aligned with my personal values.
12. I am generally satisfied with my financial compensation.
